# Assessing diagnostic performance of multimodal AI and human experts in oral and maxillofacial radiography: a comparative analysis of ChatGPT, Grok, and MANUS

**DOI:** 10.1080/07853890.2026.2664903

**Published:** 2026-05-07

**Authors:** Ahmed A. Madfa, Abdullah F. Alshammari, Bassam A. Anazi

**Affiliations:** a Department of Restorative Dental Science, College of Dentistry, University of Ha’il, Ha’il, Kingdom of Saudi Arabia; b Department of Basic Dental and Medical Science, College of Dentistry, University of Ha’il, Ha’il, Kingdom of Saudi Arabia

**Keywords:** Artificial intelligence, ChatGPT, dentistry, Grok, MANUS, large language models, radiographic diagnostics

## Abstract

**Background:**

Artificial intelligence (AI), particularly large language models (LLMs), is increasingly applied to radiographic interpretation in healthcare. In dentistry, radiographic imaging is essential for diagnosis and treatment planning, yet remains subject to variability and human error. AI may enhance diagnostic accuracy and consistency.

**Aim:**

To evaluate and compare the diagnostic accuracy, consistency, and interpretive performance of multimodal AI models—ChatGPT, Grok, and MANUS—with expert radiologists in dental radiograph interpretation.

**Methods:**

A total of 120 anonymised radiographs (40 OPGs, 40 periapical, 40 CT slices) were selected from validated academic sources. Two board-certified oral and maxillofacial radiologists established gold standard diagnoses. Each image was independently assessed by the three AI models under standardised prompting. Diagnostic accuracy and intra-model consistency were analysed using descriptive statistics, Cohen’s kappa, McNemar’s test, and logistic regression.

**Results:**

In the first assessment, MANUS and ChatGPT achieved 92.5% accuracy (111/120), while Grok reached 88.3% (106/120). Performance improved in the second round: MANUS 95.0%, ChatGPT 93.3%, and Grok 90.8%, compared with 96.7% for radiologists. ChatGPT showed the highest reproducibility (κ = 0.937), whereas MANUS demonstrated the highest overall accuracy. Strong agreement was observed between ChatGPT and MANUS, with greater variability in Grok. No significant systematic bias was detected between AI outputs and radiologist benchmarks.

**Conclusion:**

The evaluated LLMs demonstrated diagnostic performance comparable to expert radiologists. MANUS excelled in accuracy and ChatGPT in reproducibility, supporting their potential as adjunct tools in dental radiology, while maintaining the need for expert oversight.

Clinical trial number: Not applicable.

## Background

Artificial intelligence (AI) continues to transform diagnostic healthcare, with recent advances offering significant applications in dentistry. Among the most rapidly evolving tools are large language models (LLMs), which can process complex clinical information, synthesise knowledge from vast datasets, and provide real-time diagnostic reasoning [1–4]. Although these models were initially developed for language-based tasks, their capabilities have expanded into multimodal domains, including clinical image interpretation [5–8]. In dentistry, where radiographic imaging is a cornerstone of diagnostics, such innovations present new opportunities to augment clinical decision-making and reduce diagnostic variability [9,10].

Dental imaging modalities, including periapical radiographs, panoramic radiographs (also known as orthopantomograms, OPGs), and cone-beam computed tomography (CBCT), are routinely used to evaluate a wide range of oral and maxillofacial conditions [4]. From detecting periapical pathology, periodontal bone loss, and impacted teeth to assessing cystic lesions, tumours, and anatomical variations, these imaging tools provide critical information that guides diagnostics and treatment planning [5]. However, the accurate interpretation of these images requires clinical expertise and can be subject to human error, particularly in complex or ambiguous cases. The incorporation of AI tools that can support or validate radiographic interpretation could significantly enhance diagnostic accuracy and efficiency in clinical settings.

Several LLMs have emerged as promising platforms for medical applications, including ChatGPT (OpenAI), Grok (xAI), and MANUS, a model specifically designed for healthcare reasoning [6]. Although ChatGPT and Grok are primarily known for their general-purpose capabilities, recent updates have enhanced their ability to handle image-related tasks when accompanied by contextual prompts [11–14]. MANUS, in contrast, is tailored for clinical tasks and includes domain-specific training [15,16]. Despite the increasing presence of these models in healthcare dialogue, their performance in interpreting dental radiographic images, particularly across multiple modalities, remains insufficiently explored, particularly in multi-modality and human-comparative frameworks.

Although artificial intelligence has been extensively investigated in dental radiology, the majority of prior studies have focused on convolutional neural networks (CNNs) and other task-specific deep learning models for lesion detection, classification, and segmentation [17,18]. In contrast, the application of LLMs for direct radiographic interpretation remains limited and is still an emerging area of research.

Recent studies have begun exploring the performance of LLMs in medical and dental domains; however, these have primarily evaluated text-based clinical reasoning, educational tasks, or single-modality image interpretation, often without direct comparison to expert clinicians or assessment of reproducibility over time [17,18]. To date, there is a lack of comprehensive studies evaluating multimodal LLMs across different oral and maxillofacial imaging modalities using standardized diagnostic conditions.

Therefore, the present study aimed to assess the diagnostic performance, accuracy, and reproducibility of three multimodal large language models—ChatGPT, Grok, and MANUS—in interpreting oral and maxillofacial radiographic images, compared with human experts. The study evaluated each model’s ability to identify common and complex dental pathologies using a curated dataset of clinically validated images and standardized diagnostic prompts, while also assessing intra-model consistency across repeated evaluations.

Null Hypothesis of the present study stated that there is no significant difference in diagnostic accuracy, performance, or reproducibility between the multimodal AI models (ChatGPT, Grok, and MANUS) and human experts in the interpretation of dental radiographic images.

## Methods

### Ethical considerations

This study did not involve direct human participation, patient interactions, or the use of clinical images derived from live subjects. All radiological images were obtained from published academic resources, specifically textbooks widely recognised in the field of oral and maxillofacial radiology and pathology. The sources included the following: *Principles and Practice of Head and Neck Surgery and Oncology (2nd Ed.)*, *Oral and Maxillofacial Pathology (4th Ed.)*, *Contemporary Oral Oncology*, *Oral Pathology in Clinical Dental Practice*, and *Oral Medicine and Pathology at a Glance*. Given that no identifiable personal data or real patient content was used, ethical approval and consent requirements were deemed unnecessary.

### Study design

A comparative diagnostic performance evaluation was conducted to assess the interpretation capabilities of three LLMs: ChatGPT (GPT-4-turbo, OpenAI), Grok (April 2025 version, xAI), and MANUS (clinical release, April 2025). The goal was to assess their accuracy and consistency in analysing common oral and maxillofacial radiographs and scans.

### Image selection and reference standard

A total of 120 anonymised, high-resolution radiological images were selected to represent common diagnostic challenges in oral and maxillofacial radiology. The dataset included 40 OPGs, 40 periapical radiographs, and 40 CT slices. These images were systematically selected from validated academic archives and confirmed to be representative of diverse conditions, including odontogenic tumours, cysts, inflammatory lesions, osseous pathologies, and neoplasms affecting the jaw and associated structures.

### LLM interaction protocol

Each of the 120 radiographic cases was input into the three AI models under identical, controlled prompting conditions. Prompts included relevant clinical context (when appropriate), anatomical location, and a direct link or embedded static image. No follow-up clarification or adaptive coaching was provided to any of the models, ensuring uniformity.

To assess the temporal consistency of each model, the full image set was re-entered two weeks after the initial round, using the same prompts and settings. This two-week interval minimized recall bias and allowed evaluation of intra-model reproducibility. This approach enabled the comparison of responses over time within each AI system.

For each prompt, the model’s output included a diagnostic suggestion and, where available, an explanatory rationale. The primary diagnosis, defined as the most clearly indicated or prioritised term in the response, was recorded and used for evaluation.

### Human diagnostician evaluation

To benchmark the performance of LLMs against human expertise, two board-certified oral and maxillofacial radiologists, each with more than 8 years of diagnostic experience, were independently asked to interpret the same 120 digital radiographic cases (40 OPGs, 40 periapical radiographs, and 40 CT slices) used in the AI evaluation. The selection of experienced specialists was intended to ensure a consistent and high-level reference standard, thereby minimising variability related to differences in clinical experience. Diagnoses were recorded without access to AI outputs or peer input. Each radiologist was instructed to provide a single primary diagnosis per case. After completing their independent assessments, both radiologists met to review and resolve any discrepancies, reaching a consensus diagnosis for each case. These finalised diagnoses served as the reference standard against which diagnostic accuracy and interobserver agreement were evaluated. Agreement between the two radiologists was quantified using descriptive statistics and Cohen’s kappa coefficient.

### Evaluation criteria

**Figure 1.**
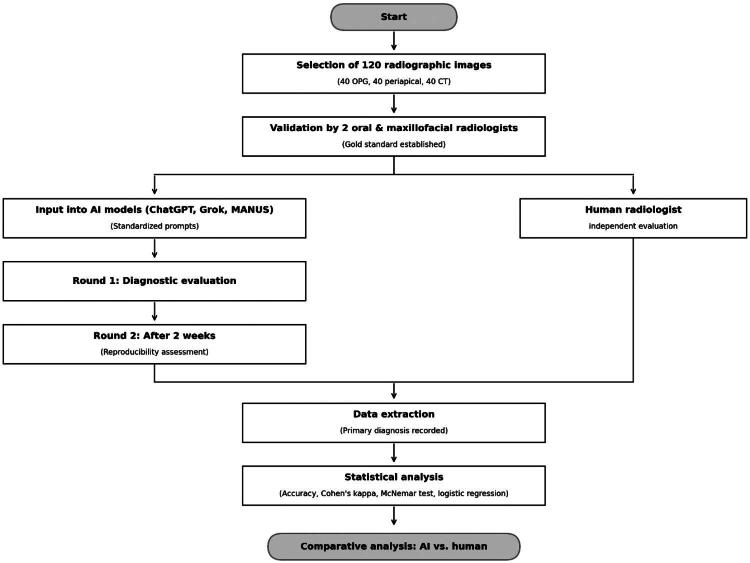
Flowchart illustrating the study design and workflow, including image selection, reference standard establishment, AI model evaluation, human expert assessment, and statistical analysis.

The overall study design and workflow are summarised in Figure 1, illustrating the processes of image selection, AI evaluation, human expert assessment, and statistical analysis.

The primary performance metric was diagnostic accuracy, defined as the proportion of cases in which the model-generated primary diagnosis exactly matched the reference standard established by expert radiologists. Each response was categorised as either correct or incorrect. Evaluation was performed independently by two expert reviewers. Inter-rater reliability was calculated using Cohen’s kappa, targeting a κ value of 0.85 or higher to ensure consistent scoring. Disagreements were addressed through discussion and mutual agreement. In addition, intra-model diagnostic consistency was assessed by comparing each model’s outputs from the first and second rounds. Concordance rates were quantified using Cohen’s kappa to determine the stability of diagnostic reasoning over time.

### Statistical analysis

Descriptive statistics were used to summarise diagnostic accuracy and error rates for each AI model and human radiologists. Intra-model consistency and inter-rater agreement with human experts were evaluated using Cohen’s kappa (κ). Paired comparisons of diagnostic outcomes were performed using McNemar’s test to identify significant differences in accuracy between AI models and between AI and human diagnoses. Binary logistic regression assessed whether AI outputs systematically deviated from the human reference standard, reporting odds ratios with 95% confidence intervals. Overall performance variation among the AI models was further evaluated using chi-square tests where applicable. Statistical significance was defined as *p* < 0.05.

## Results

### Overall diagnostic accuracy

**Figure 2.**
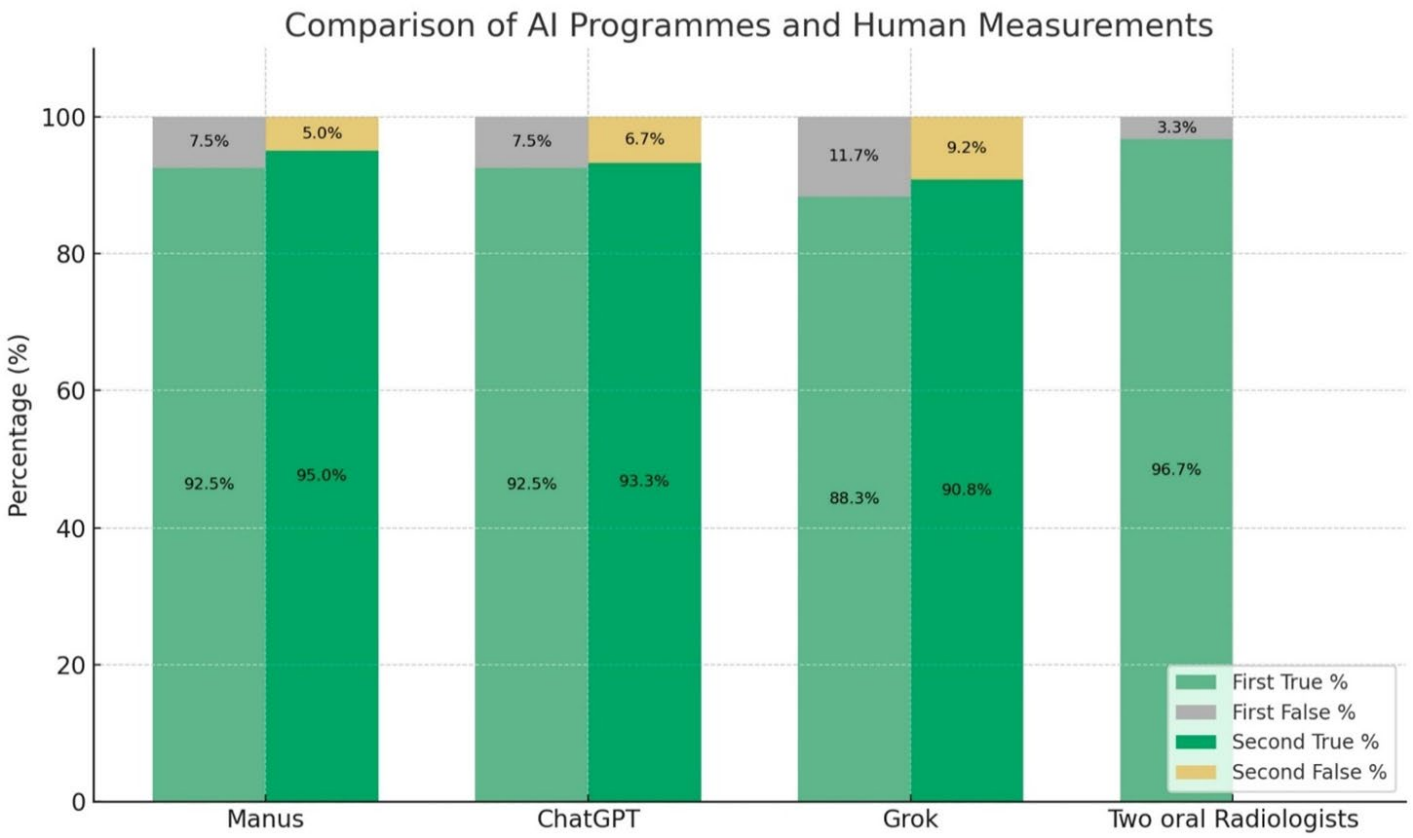
Comparative diagnostic accuracy of human experts and AI Models (ChatGPT, Grok, and MANUS) in interpreting dental radiographs across two assessment rounds.

The diagnostic performance of the three LLMs (MANUS, ChatGPT, and Grok) was evaluated against the diagnoses of two board-certified oral and maxillofacial radiologists, who served as the human reference benchmark. Of the 120 radiographic cases, MANUS and ChatGPT each correctly diagnosed 111 cases in the first round, yielding an accuracy of 92.5%. Grok achieved slightly lower performance, with 106 correct classifications (88.3%). In the second round of assessment, diagnostic accuracy improved across all models: MANUS achieved 114/120 (95.0%), ChatGPT reached 112/120 (93.3%), and Grok attained 109/120 (90.8%). In contrast, the radiologists demonstrated the highest overall accuracy, with 116 out of 120 (96.7%) correct classifications. Misclassification rates followed an inverse trend: Grok exhibited the highest error proportion (11.7% in the first round and 9.2% in the second), while radiologists recorded only four errors (3.3%). These comparative results are presented in Table 1 and Figure 2.

**Table 1.** Comparison between human measurement and various answers of AI programmes.

Variables	First measurement *n* (%)		Second measurement *n* (%)	
	True answers	False answers	True answers	False answers
**MANUS**	111 (92.5%)	9 (7.5%)	114 (95.0%)	6 (5.0%)
**ChatGPT**	111 (92.5%)	9 (7.5%)	112 (93.3%)	8 (6.7%)
**Grok**	106 (88.3%)	14 (11.7%)	109 (90.8%)	11 (9.2%)
**Two oral Radiologists**	116 (96.7%)	4 (3.3%)	–	–

### Intra-model consistency

Consistency between first- and second-round assessments was quantified using Cohen’s kappa coefficients and McNemar’s test. ChatGPT demonstrated the highest temporal reproducibility, with a κ value of 0.937, indicating almost perfect agreement between rounds. McNemar’s test confirmed no significant difference across rounds (*p* = 1.000), underscoring the stability of ChatGPT’s diagnostic reasoning. Grok also showed strong reproducibility, with a κ of 0.866 and no significant difference between rounds (*p* = 0.250). MANUS demonstrated moderate-to-substantial agreement, with a κ of 0.787, with no significant round-to-round variation (*p* = 0.250). The intra-observer reproducibility could not be calculated for the radiologists since only a single round of independent interpretation was performed. The aforementioned findings, detailed in Table 2, highlight the trade-off between accuracy and consistency, with MANUS achieving the highest overall accuracy and ChatGPT demonstrating the most stable diagnostic behaviour.

**Table 2.** Intra-model consistency between first and second measurements.

Manus			
Measurement		Second measurement	
		Correct	Incorrect
Frist measurement	Correct	111 (92.5%)	9 (7.5%)
	Incorrect	114 (95.0%)	6 (5.0%)
Measure of agreement kappa	0.787		
McNemar test	0.250		
ChatGPT			
Measurement		Second measurement	
		Correct	Incorrect
Frist measurement	Correct	111 (92.5%)	9 (7.5%)
	Incorrect	112 (93.3%)	8 (6.7%)
Measure of agreement kappa	0.937		
McNemar test	1.000		
Grok			
Measurement		Second measurement	
		Correct	Incorrect
Frist measurement	Correct	106 (88.3%)	14 (11.7%)
	Incorrect	109 (90.8%)	11 (9.2%)
Measure of agreement kappa	0.866		
McNemar test	0.250		

### Pairwise inter-model comparisons

Direct pairwise comparisons between AI models revealed distinct patterns of agreement and divergence. MANUS and ChatGPT were generally aligned, with no significant differences in diagnostic outputs across rounds (*p* values ranging from 0.375 to 1.000). For example, in the second round, ChatGPT and MANUS produced concordant results in 110 cases, differing in only a handful of instances. In contrast, Grok’s performance diverged more sharply, particularly when compared with MANUS, where the second-round comparison reached significance (*p* = 0.021). Similarly, Grok showed borderline divergence from ChatGPT (*p* = 0.070).

No significant differences were observed when AI models were compared against the human benchmark. Radiologists achieved near-perfect alignment with MANUS (*p* = 0.562), ChatGPT (*p* = 0.562), and Grok (*p* = 0.398), suggesting that although subtle discrepancies occurred, the overall diagnostic agreement between humans and AI was consistently high. These inter-model and human–AI comparative outcomes are detailed in Table 3.

**Table 3.** Pairwise comparison of diagnostic agreement between human measurement and various AI programmes.

AI models		Grok			ChatGPT			Manus		
		Correct	Incorrect	*P* value	Correct	Incorrect	*P* value	Correct	Incorrect	*P* value
Manus	Correct	106	5	0.727	108	3	1.000			
	Incorrect	3	6	–	4	5	–			
ChatGPT	Correct	107	4	0.687				110	1	0.375
	Incorrect	2	7	–				4	5	–
Grok	Correct				105	7	0.070	105	9	0.021
	Incorrect				1	7	–	1	5	
Two oral radiologists	Correct	1	3	0.398	0	4	0.562	0	4	0.562
	Incorrect	103	13	–	107	9	–	107	9	–

### Human vs. AI diagnostic patterns

Although all of the AI models demonstrated performance approaching expert human levels, the radiologists consistently outperformed the models by a modest margin (Figure 2). Importantly, radiologists committed only four errors, compared with 6–14 errors in the first round of AI assessments. The discrepancy narrowed in the second round, particularly for MANUS (6 errors) and ChatGPT (8 errors). This progression highlights the potential for AI models to achieve human-level diagnostic accuracy in radiographic interpretation, although differences persist in fine-grained diagnostic reasoning.

### Logistic regression analysis

Binary logistic regression was conducted to evaluate whether the diagnostic outcomes of AI models systematically deviated from those of radiologists. For MANUS, the regression coefficient (B) was −0.367 (SE = 0.437, *p* = 0.400), producing an odds ratio (Exp(B)) of 0.693 with a 95% confidence interval (0.294–1.630). ChatGPT demonstrated complete neutrality, with *B* = 0.000 (SE = 0.424, *p* = 1.000) and an odds ratio of 1.000 (95% CI: 0.435–2.298). Grok yielded *B* = −0.122 (SE = 0.349, *p* = 0.728), with an odds ratio of 0.886 (95% CI: 0.447–1.756). None of these results were significant, confirming that the AI models did not exhibit systematic bias relative to the radiologists. These regression outputs are summarised in Table 4.

**Table 4.** Comparison between two oral radiologists and various AI programmes.

Variables		*B*	SE	Wald	*df*	Sig.	Exp (B)	Lower	Upper
MANUS	Radiograph	−.367-	0.437	0.707	1	.400	.693	0.294	1.630
ChatGPT	Radiograph	0.000	0.424	0.000	1	1.000	1.000	0.435	2.298
Grok	Radiograph	−0.122-	0.349	0.121	1	0.728	0.886	0.447	1.756

### Influence of radiographic modality

Binary logistic regression analysis was performed to evaluate whether the type of radiographic image influenced diagnostic performance (Table 5). The results demonstrated that the type of radiograph was not a statistically significant predictor of diagnostic accuracy (*B* = 0.397, SE = 0.646, Wald = 0.379, *p* = 0.538, Exp(B) = 1.488, 95% CI: 0.420–5.277). These findings indicate that diagnostic performance of the evaluated AI models remained consistent across different imaging modalities, including periapical, panoramic, and CT images.

**Table 5.** Binary logistic regression analysis for diagnostic performance by type of radiographic image.

Variables	*B*	SE	Wald	*df*	Sig.	Exp (B)	Lower	Upper
Type of radiograph	0.397	0.397	0.397	1	0.538	1.488	0.420	5.277

## Discussion

This study provides compelling evidence that LLMs can achieve diagnostic performance approaching that of board-certified oral and maxillofacial radiologists when interpreting dental radiographs. The null hypothesis stated that no significant difference exists in diagnostic accuracy, performance, or reproducibility between the multimodal AI models (ChatGPT, Grok, and MANUS) and human experts in interpreting dental radiographic images. Statistical analyses supported this hypothesis, as McNemar’s test and logistic regression revealed no significant discrepancies or systematic bias between AI and human diagnoses. Although MANUS achieved the highest accuracy and ChatGPT demonstrated the greatest reproducibility, these differences were not statistically significant. Therefore, the null hypothesis was accepted, indicating comparable diagnostic performance between AI models and expert radiologists within the study parameters.

Recent advances in artificial intelligence have increasingly demonstrated the utility of large language models across medical and scientific domains, including radiographic interpretation and hybrid analytical frameworks that integrate traditional diagnostic approaches with computational reasoning. For example, recent studies have highlighted how LLM-based systems can be applied not only for clinical interpretation but also for supporting experimental modelling and theoretical problem-solving across disciplines, reinforcing their versatility beyond purely linguistic tasks [19**–**21]. These developments align with the present findings, where multimodal LLMs demonstrated reliable performance in structured radiographic interpretation tasks, suggesting that such systems may increasingly serve as adjuncts in diagnostic workflows rather than isolated experimental tools.

In the initial assessment round, MANUS and ChatGPT correctly classified 92.5% of cases, while Grok achieved an accuracy of 88.3%. Upon re-evaluation, all models demonstrated improved performance. The human benchmark was 96.7%; MANUS reached 95.0%, ChatGPT 93.3%, and Grok 90.8%. The observed difference between the highest-performing LLM (MANUS) and expert radiologists was 1.7 percentage points (95.0% vs. 96.7%). Although this gap is small in absolute terms, it should not be interpreted as statistical equivalence, as equivalence or non-inferiority testing was not conducted. Nevertheless, the findings suggest that the performance of advanced LLMs may be approaching expert-level accuracy in dental imaging tasks such as caries detection and periapical lesion identification [22–26]. From a clinical perspective, even small diagnostic errors remain important; false positives may lead to unnecessary interventions, whereas false negatives may result in missed or delayed diagnosis of significant pathology. Therefore, despite high overall accuracy, careful clinical interpretation and expert oversight remain essential.

The improvements observed in the second evaluation round may reflect iterative reasoning prompts or enhanced model familiarity with the diagnostic task, consistent with ‘few-shot adaptation’ phenomena [26]. Nonetheless, the persistent superiority of human radiologists underscores the indispensable value of clinical experience, particularly in borderline or atypical cases, where nuanced pattern recognition and contextual understanding are crucial [22–25]. Misclassification analysis suggested that Grok’s relatively higher error rate may have resulted from differences in training data or reasoning architecture, emphasising the importance of rigorous domain-specific fine-tuning.

From a clinical perspective, the high accuracy of MANUS and ChatGPT suggests their potential utility as decision-support tools, either by providing preliminary reads or serving as a secondary opinion to improve workflow efficiency. However, safe implementation requires regulatory oversight, ongoing validation on diverse datasets, and attention to medico-legal considerations [27,28]. Consequently, these AI systems should complement, rather than replace, expert interpretation.

Although this study demonstrated high overall diagnostic accuracy, the underlying reasons for AI misdiagnoses were not systematically analysed. This represents an important consideration, as errors may arise from factors such as image complexity, ambiguous radiographic features, and limited clinical context, which are well-recognised challenges in dental radiographic interpretation [22,28]. In addition, differences in model architecture and training data may further contribute to variability in diagnostic performance. Importantly, these models rely on probabilistic pattern recognition rather than true clinical reasoning, which may affect performance in borderline cases. Future studies incorporating detailed, case-level error analysis are needed to better understand these misdiagnosis patterns and improve model reliability.

Beyond accuracy, LLMs demonstrated high reproducibility and strong agreement with human radiologists. ChatGPT exhibited the most stable round-to-round performance, with an almost perfect Cohen’s kappa of 0.937 and no significant McNemar difference (*p* = 1.000). Grok also showed strong reproducibility (κ = 0.866), whereas MANUS, despite achieving the highest absolute accuracy, displayed slightly lower temporal consistency (κ = 0.787). These findings reinforce prior evidence that consistency is a distinct but essential metric in evaluating clinical AI systems [29–32]. While the inclusion of a 2-week reassessment provides insight into short-term reproducibility, evaluation of long-term performance remains an important area for future research, particularly given the evolving nature of AI models.

Pairwise comparisons revealed that MANUS and ChatGPT were highly concordant, differing in only a few cases, whereas Grok diverged more frequently, especially relative to MANUS (second-round *p* = 0.021). Such variability likely reflects differences in model architecture or training corpora, consistent with observations that foundation models can vary widely in domain robustness despite similar headline accuracies [33]. Crucially, all AI–human comparisons yielded non-significant McNemar results, and logistic regression confirmed the absence of systematic bias: the odds of disagreement between each LLM and the radiologists approximated unity. This lack of bias is pivotal for clinical adoption, aligning with recommendations for ‘equivalence testing’ when evaluating AI as a clinical adjunct [34].

Although radiologists maintained a modest accuracy advantage (96.7% versus 90.8–95.0%), the narrowing gap—particularly for MANUS and ChatGPT—underscores the adaptive potential of LLMs. Iterative prompting and contextual reasoning may explain these improvements; however, nuanced clinical interpretation, especially in borderline or mixed-pathology cases, continues to favour experienced clinicians [26,33].

From a translational standpoint, the combination of high accuracy, reproducibility, and minimal AI–human bias suggests that these models could serve as reliable decision-support tools in oral and maxillofacial radiology. Potential applications include preliminary triage, double reading for quality assurance, and educational support for trainees. Nevertheless, prospective multi-centre validation and stringent regulatory oversight are prerequisites for safe clinical deployment [27]. As this study focused on radiographic image interpretation rather than patient-level variables, the impact of demographic diversity on AI performance was not evaluated and warrants further investigation in future studies.

Overall, this study highlights the potential of contemporary LLMs to perform dental radiographic interpretation with a level of diagnostic accuracy and temporal reproducibility that approaches that of board-certified oral and maxillofacial radiologists. Their consistently high concordance with expert assessments suggests several valuable clinical applications. LLMs could function as decision-support tools by providing preliminary interpretations to guide workflow prioritisation or triage, or by offering rapid second opinions to assist clinicians in complex or borderline cases. These models may also serve as interactive educational resources for dental trainees, helping to standardise teaching and reduce inter-observer variability. Importantly, the absence of detectable systematic bias when compared with human radiologists supports the safe deployment of these systems as adjuncts within a supervised clinical environment. Nevertheless, these systems are intended to complement, not replace, expert judgment; final diagnostic responsibility must remain with licensed radiologists or appropriately credentialed dental professionals.

The use of AI in clinical practice raises important ethical considerations, including accountability, transparency, and potential bias. AI should support—not replace—clinical judgment, with responsibility remaining with qualified professionals. Clear regulatory and ethical frameworks are essential for safe implementation.

Several limitations should be acknowledged. First, the retrospective design and relatively small sample of 120 radiographs limit generalisability to broader patient populations and imaging conditions. In addition, the use of radiographic images sourced from validated academic archives may introduce selection bias, as such datasets typically contain well-defined, high-quality, and diagnostically representative cases with reduced variability. This may lead to an overestimation of diagnostic performance compared with real-world clinical settings, where images often include artefacts, suboptimal exposures, and greater anatomical and pathological complexity. While this design enabled controlled and standardised comparisons, future studies should incorporate prospective, real-world clinical datasets to improve generalisability. Second, only two radiologists served as the human benchmark, and intra-observer variability could not be assessed. Furthermore, although consensus between two experienced oral and maxillofacial radiologists was used to establish the reference standard, this approach may still be subject to inherent human variability and diagnostic subjectivity. Third, the analysis evaluated fixed versions of the models under a specific prompting strategy; future software updates or alternative prompting approaches may yield different results. Prospective, multi-institutional studies with larger and more diverse datasets are needed to validate these findings and determine the real-world clinical impact of LLM-assisted radiographic interpretation.

## Conclusions

Large language models demonstrated diagnostic accuracy and reproducibility that closely approached the performance of board-certified oral and maxillofacial radiologists in interpreting dental radiographs. MANUS achieved the highest overall accuracy, while ChatGPT exhibited the greatest temporal consistency; all models showed strong agreement with human experts, without evidence of systematic bias. These findings highlight the potential of LLMs as reliable adjuncts for clinical decision support, quality assurance, and educational applications in oral and maxillofacial radiology. Nevertheless, broader validation on multicentre datasets and careful regulatory oversight are essential before these tools can be adopted for routine patient care.

## Data Availability

The datasets created and/or analyzed for the current study are not publicly accessible because ethics approval was given on the grounds that only the researchers involved in the study would have access to the identified data, but they are available from the corresponding author upon justifiable request.
